# Th1/Th2 polarization of peripheral immune response in atherothrombotic and cardioembolic stroke: a prospective study

**DOI:** 10.1038/s41598-022-20515-x

**Published:** 2022-09-30

**Authors:** Simone Bellavia, Irene Scala, Pier Andrea Rizzo, Valerio Brunetti, Aldobrando Broccolini, Giacomo Della Marca, Paolo Calabresi, Giovanni Frisullo

**Affiliations:** 1 Neurologia, Università Cattolica del Sacro Cuore, Rome, Italy; 2 UOC Neurologia, Dipartimento di Scienze dell’invecchiamento, Neurologiche, Ortopediche e della Testa-Collo, Fondazione Policlinico Universitario “A Gemelli, IRCCS, Largo Agostino Gemelli, 8, 00168 Rome, Italy

**Keywords:** Cell biology, Immunology, Neuroscience

## Abstract

Compelling evidence suggest a key role of immune system in the development and progression of ischemic stroke. Although the balance between proinflammatory CD4 + T helper (Th)-1 lymphocytes, expressing T-bet transcription factor, and anti-inflammatory Th2 cells expressing GATA3 seems to influence the outcome in experimental stroke, the role of peripheral immune response in acute stroke patients is poorly understood. We aimed to evaluate the peripheral Th1/Th2 balance in acute atherothrombotic (ATHS) and cardioembolic stroke (CES) patients and in age- and sex-matched healthy subjects. Using flow cytometry, we analyzed the percentage of CD4 + T-bet + T cells and CD4 + GATA3 + T cells from peripheral blood of ATHS and CES patients (2,4 and 7 days after stroke onset). Patients and controls were screened for infectious conditions, autoimmune, inflammatory, or cancerous diseases. On day 2 circulating CD4 + T-bet + T cells were significantly higher in stroke patients compared to controls, and in ATHS compared to CES and controls. On day 7, we observed a significant increase of CD4 + T-bet + T cells in both ATHS and CES patients compared to baseline. No difference was observed in circulating CD4 + GATA3 + T cells among ATHS, CES patients, and controls. These data suggest that circulating CD4 + T-bet + T cells could be useful marker indicating atherothrombotic genesis of stroke and provide new insight into the peripheral adaptive immune response in acute stroke.

## Introduction

Stroke is a serious life-threatening medical condition that is the second leading cause of death and a major cause of disability worldwide^1^. Ischemic stroke (IS) typically results from a thrombotic or thromboembolic blockage of a cerebral artery which leads to a drastic decrease in the brain's blood supply, however to reduce its pathophysiology to an hydraulic problem would be simplistic^2^. It is well known that a pre-existing systemic inflammatory condition, like an autoimmune or neoplastic disease, can contributes to the onset of IS^3^, but recent studies outlines how the interaction between stroke and inflammation could be even more complex. In fact, brain ischemia triggers and, at the same time, is influenced by a wide array of secondary processes, including the local and systemic inflammation able to influence the development and progression of ischemic damage^4,5^. Following IS, resident microglia and circulating components of the innate immunity represent the first line of response to ischemia^6,7^ but damaged brain tissue also exposes and releases cryptic epitopes, which are presented to the adaptive immunity in cervical lymph nodes^8^. It results in a dysregulation of immune response with the induction both of a systemic immunosuppression which increases the infectious risk^9^, and of autoimmune response which may further exacerbate brain injury and worsen long-term outcome^10,11^. Despite the antigen-mediated activation of acquired immunity could influence cerebral ischemia outcome, compelling evidence from animal models shows how the detrimental role played by T lymphocytes can be mediated by a non-antigen dependent activation^12^.

Evaluating adaptive immunity and T cell subsets infiltrating the injured brain in experimental stroke models, T helper type 1 (Th1)/Th2 balance seems to play a watershed role on the evolution of ischemic brain injury^12,13^. In particular, Th1 cells, a lineage of CD4 + effector T cells expressing the key regulator transcription factor T-bet^14^, promote the production of proinflammatory cytokines, chemokines and reactive oxygen species and the disruption of the blood brain barrier, inducing ischemic lesion progression^15,16^. On the other side, Th2 cells, CD4 + effector T cells, controlled by the transcription factor GATA3^14^, promote debris removal, tissue remodeling, and repair after brain ischemia, secreting anti-inflammatory cytokines, and inducing the production of nerve growth factor^16^. The Th1/Th2 balance could also directly influence the ischemic lesion volume as shown in IL-4 knockout mice in which an increase in the Th1/Th2 ratio is associated both with worse neurological outcomes and with larger infarction than wild-type mice^17^. Although in vitro and in vivo experimental studies pointed out the impact of peripheral immune response on ischemic brain injury and stroke evolution^16^, the role of Th1/Th2 balance in peripheral blood of IS patients has been poorly addressed and not completely understood. The aim of our study is to assess the balance between Type-1 and Type-2 polarization in acute stroke, evaluating the expression of T-bet and GATA3 in CD4 + T cells in peripheral blood of acute stroke patients, comparing them to healthy subjects. Moreover, to better discriminate the pre-existing pro-inflammatory immune response, already described in patients with atherosclerotic disease, from the secondary response to ischemic damage^18,19^, we decided to evaluate the Th1/Th2 balance by comparing patients with atherothrombotic stroke compared to patients with cardioembolic stroke.

## Materials and methods

### Study design and population

In this observational study, we included all consecutive patients affected by IS, admitted to Neurological sub-intensive care-Unit, over a period of 6 months. During the hospitalization, medical history, reporting demographic data, vascular risk factors, previous inflammatory, autoimmune or cancer diseases, atrial fibrillation, and the National Institute of Health Stroke Scale (NIHSS) were collected. IS etiology was determined according to the trial of ORG 10,172 in acute stroke treatment (TOAST) classification^20^. Inclusion criteria were: (1) IS confirmed by CT scan and/or brain MRI during hospitalization; (2) large-artery atherosclerosis or cardioembolism according to TOAST criteria; (3) age ≥ 18 years; (4) providing signed and dated informed consent. Exclusion criteria were: (1) small-vessel occlusion; (2) intracranial hemorrhage; (3) wake-up stroke; (4) thrombolysis or thrombectomy; (5) any infection in the last 30 days from the IS onset; (6) medical history of autoimmune, inflammatory, or cancer disease; (7) ongoing treatment with steroids, immunosuppressive and immunomodulatory drugs or antibiotics or during the last 30 days before admission; (8) pregnancy; (9) stroke of undetermined etiology to minimize the risk of underlying undiagnosed inflammatory or autoimmune disease. The last exclusion criterion was necessary to minimize the risk of including underlying undiagnosed inflammatory or autoimmune disease. Patients who developed a clinical or laboratory infection during the first 8 days of hospitalization were excluded from the study. As control group, we included age- and sex-matched healthy subjects who were screened for ongoing or previous infectious conditions, autoimmune, inflammatory or cancer diseases. Blood samples for immunological analysis were collected within 48 h, and at 4 and 7 days from stroke onset. Healthy subjects were subjected to a single blood sample. This study was approved by the Ethics Committee of Fondazione Policlinico Universitario “A.Gemelli” (study ID: 4823). All methods were performed in accordance with the relevant guidelines and regulations.

### Intracellular flow cytometric analysis

Peripheral blood mononuclear cells were isolated from peripheral blood by density gradient centrifugation over a Ficoll–Hypaque density gradient and analyzed using a double labeling procedure staining with anti-CD4-PE-Cy5 antibodies (Beckman Coulter, Miami, USA), followed by fixation, permeabilization, and incubation with PE anti-T-bet antibody and PE anti-GATA3 antibody (Biolegend, San Diego, USA). The analysis was performed by a Coulter Epics-XL-MCL flow cytometer (Coulter, Miami, FL, USA). For the detection of T-bet and GATA-3 in CD4 + T cells, PBMCs were analyzed using a double labeling procedure staining with anti-CD4-PE-Cy5 antibodies (Beckman Coulter, Miami, FL, U SA), followed by fixation, permeabilization, and incubation with PE anti-T-bet Antibody and PE anti-GATA3 Antibody (Biolegend, 8999 BioLegend Way San Diego, CA 92121). Appropriate fluorochrome-conjugated isotype-matched mAbs (PE Mouse IgG1, κ Isotype Ctrl, PE Mouse IgG2b, κ Isotype Ctrl—Biolegend) were used as control for background staining in each flow acquisition. Each analysis was performed using at least 50,000 cells that were gated in the region of the lymphocyte population, as determined by light scatter properties (forward scatter versus side scatter). To analyze the expression of intracellar tanscription factor (T-bet and GATA3), lymphocyte population were initially gated in in a forward- and side-scatter gate. A second gate was then created around the CD4 + cells in a CD4 + versus SSC dot plot. The percentages of T-bet/GATA-3 expressing cells were calculated in the total CD4 + population and not in the total T cell population. Quadrants of dot plot were set using appropriate isotype controls for each intra- and extracellular antibody. Researcher gating the cytofluorimetric analysis was blind to the identity of the subjects.

### Statistical analysis

Statistical analyses were performed using Statistical Package for Social Science software version 20. The significance level was set at p = 0.05 and statistical tests were 2-sided. We used the Kolmogorov–Smirnov test to evaluate the distribution of data. Mann–Whitney U-test with exact significance was used when Kolmogorov–Smirnov test indicated a non-normal distribution. Independent t-test was used for comparison of continuous variables with normally distributed data. Dichotomous variables were compared using Fisher’ exact test. Ordinal variables were analyzed with the Wilcoxon rank-sum test with exact testing. Given the exploratory nature of this study and the fact that no clinical a-priori hypothesis was available, no sample size analysis was performed.

### Ethical approval and informed consent

This study protocol was approved reviewed and approved by Ethics Committee of Fondazione Policlinico Universitario “A Gemelli”. Written informed consent was obtained from participants to participate in the study.

## Results

### Patients: demographic and clinical features

From 342 patients with acute IS assessed for the study eligibility, 32 patients affected by acute atherothrombotic stroke (ATHS, n = 18) or cardioembolic stroke (CES, n = 14) were included in the study (Flow diagram in Supplementary Fig. S1). We did not find significant differences in age and sex among ATHS, CES, and control group (n = 30). Cerebrovascular risk factors were also uniformly distributed among the study groups. Table 1 reports demographic and clinical features of our patient population and controls.

**Table 1:** Demographic features, risk factors, stroke classification, clinical assessment, and procedures of ATHS, CES and control group.

Demographic features and risk factors	ATHS (n = 18)	CES (n = 14)	Controls (n = 30)
Age, median (yr, IQR)^‡^	69.2 (11)	73.6 (10)	71.2 (10)
Gender, female, n (%)^†^	11 (61)	7 (50)	19 (63)
**Risk Factors**			
Hypertension, n (%)^†^	15 (83)	11 (79)	22 (73)
Diabetes mellitus, n (%)^†^	10 (56)	9 (64)	15 (50)
Hyperlipidaemia, n (%)^†^	8 (44)	7 (50)	11 (37)
Cigarette smoking, n (%)^†^	7 (39)	6 (42)	11 (37)
**Treatments**			
Antiplatelet therapy prior to stroke, n (%)^†^	10 (56)^1^	4 (29)^1^	11 (37)
Anticoagulant therapy prior to stroke, n (%)^†^	0 (0)^1^	9 (64)^1,2^	0 (0)^2^
**OCSP**^**1**^** classification**			
ACI, n (%)^†^	9 (50)	7 (50)	NA
POCI, n (%)^†^	3 (17)	3 (21)	NA
LACI, n (%)^†^	0 (0)	0 (0)	NA
TACI, n (%)^†^	6 (33)	4 (0)	NA
**Clinical assessment and procedures**			
NIHSS at stroke onset, median (IQR)^§^	12.4 (8)	11.8 (9)	NA
NIHSS after discharge, median (IQR)^§^	4.2 (6)	2 (5)	NA
GCS at stroke onset, median (IQR)^§^	14 (2)	15 (1)	NA

Categorical variables are expressed as number (n) and percentage (%). Numerical variables are expressed as median ± IQR.

*OSCP* The Oxfordshire Community Stroke Project, *PACI* partial anterior cerebral infarction, *POCI* posterior cerebral infarction, *LACI* lacunar cerebral infarction, *TACI* total anterior cerebral infarction, *NIHSS* National Institute of Health Stroke Scale, *GCS* Glasgow Coma Scale.

^1,2^ p < 0.05.

^†^Fisher exact test.

^‡^Wilcoxon rank-sum test.

^§^Mann–Whitney U test.

### T-bet + CD4 + T cells and GATA3 + CD4 + T cells in stroke patients and controls

**Figure 1:**
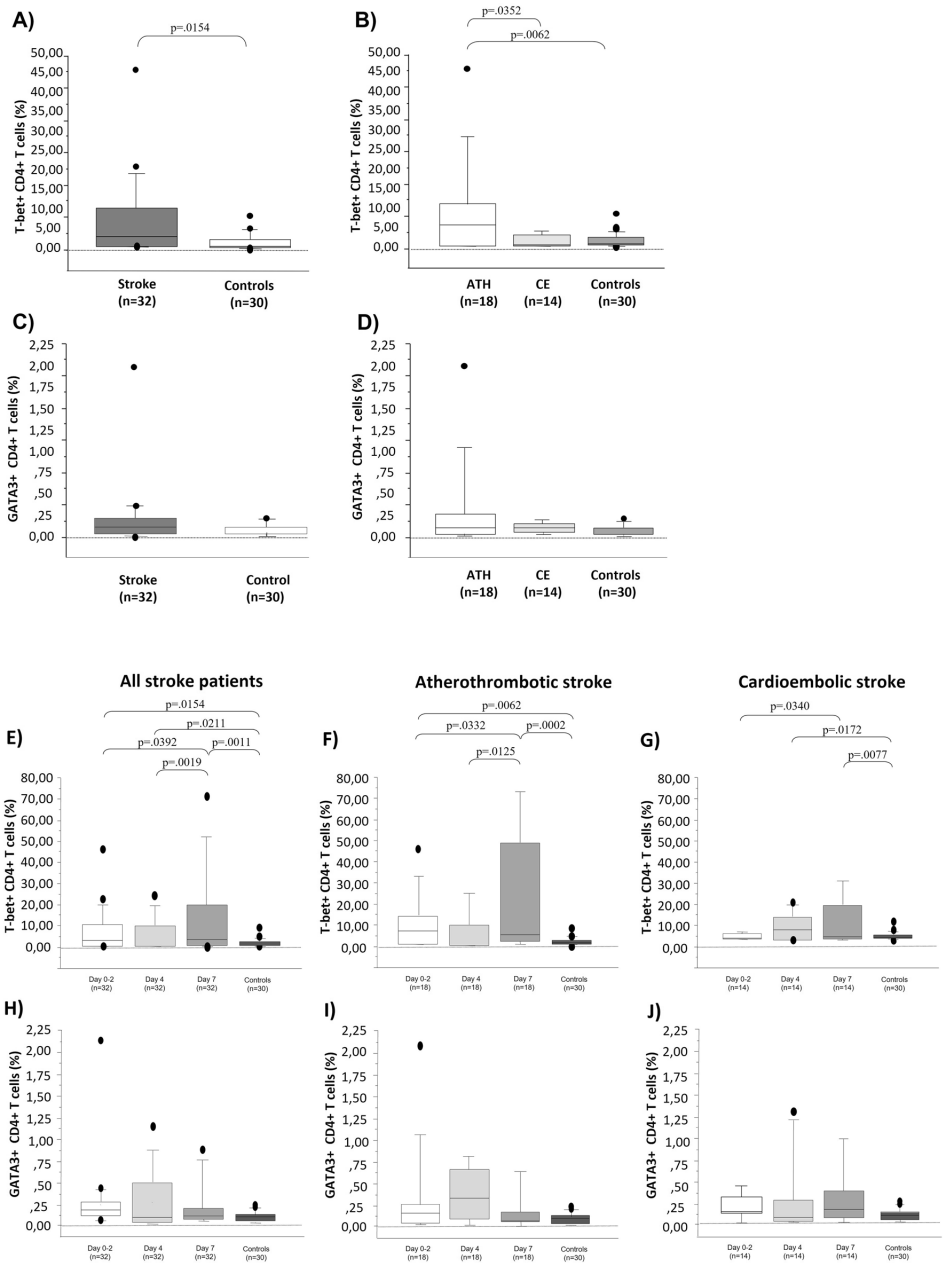
Percentage of circulating CD4 + T-bet + (**A**,**B**,**E**–**G**) and CD4 + GATA3 + T cells (**C**,**D**,**H**–**L**) in acute stroke patients and controls. Box plots express the first (Q1) and third (Q3) quartiles within a given dataset by the upper and lower horizontal lines in a rectangular box, in which there is a horizontal line showing the median. The whiskers extend upwards and downwards to the highest or lowest observation within the upper (Q3 + 1.5 × interquartile range) and lower (Q1 − 1.5 × interquartile range) limits. Data points that are outside this interval are represented as points on the graph and considered potential outliers. *p* values indicate statistical significances (< 0.05) between the different groups.

Evaluating circulating Th1 percentage in peripheral blood on day 2, we observed a significant higher percentage of CD4 + T-bet + T cells in stroke patients compared to controls (p = 0.0154; Fig. 1A). Considering stroke etiopathogenesis, we observed a higher percentage of CD4 + T-bet + T cells in peripheral blood from ATHS patients than CES patients and controls (*p* = 0.0352 and *p* = 0.0062, respectively; shown in Fig. 1B). Evaluating circulating Th2 cells, no statistically significant difference was instead observed in the percentage of GATA3 + Th2 cells in the peripheral blood of IS patients compared to controls. Moreover, we did not observe a significant difference in circulating Th2 cells among ATHS patients, CES patients, and controls (shown in Fig. 1C,D).

### T-bet + CD4 + T cells and GATA3 + CD4 + T cells in stroke patients during the first week after IS

Circulating Th1 cells were significantly increased during the first week after acute stroke. In all stroke patients the percentage of circulating CD4 + T-bet + T cells on 7 days from stroke onset was significantly higher than on day 2 and 4, and controls (*p* = 0.0392, *p* = 0.0019 and *p* = 0.0011, respectively; shown in Fig. 1E). In particular, considering pathogenic IS phenotypes, the percentage of CD4 + T-bet + T cells was significantly higher after 7 days from stroke onset than baseline both in ATHS and CES patients (*p* = 0.0332 and *p* = 0.0340, respectively; shown in Fig. 1F,G). Instead, no statistically significant difference was found at any time point in the percentage of circulating CD4 + GATA3 + T cells in the peripheral blood of patients compared to controls (shown in Fig. 1H–L).

## Discussion

In this study we evaluated the peripheral immunological response to cerebral ischemia thought the analysis of the Th1/Th2 balance, and we found a shift towards the Th1 phenotype. The role of the immune system in stroke was neglected for years until the introduction of promising neuroprotective therapies highlighted the possible role of inflammation in sparing brain tissue^21^.

It has become increasingly evident that stroke induces an acute immune response that engages and, at the same time, is engaged by local and peripheral immunological compartments. While many studies in humans and rodents have focused on the local immune response within or next to the ischemic brain injury demonstrating the involvement of resident brain cells^22,23^, as astrocytes and microglia, and invading immune cells, as neutrophils, monocytes and lymphocytes^16^, the peripheral immune response has been poorly characterized, particularly in stroke human patients^24^. An increase of non-specific serum biomarkers as white blood cell count, fibrinogen, D-dimer, and C-reactive protein as well as of serum cytokines as interleukin (IL)-1β, IL-4, IL-6, TL-12, IL-17, IL-23, IL-34, IL-37, TNF-alfa has been widely described as a systemic inflammatory response to ischemic brain damage^25,26^. Nevertheless, only few conflicting data are available about the polarization of peripheral immune cells in patients with acute stroke in the first hours after the onset of brain ischemia showing a decrease^27^ or an increase^28^ of regulatory T (Treg) cells and a decrease of B cells, Th cells, cytotoxic T cells, and NK cells in stroke patients on days 1, 3, and 7^28^. Except for the fluctuation of Treg cells, there are no data on the Th1/Th2 balance in the peripheral blood of acute IS patients.

In our study, we observed a significant increase in circulating CD4 + T-bet + T cells, expression of an involvement of Th1 response, in very early stages of cerebral ischemia which tended to increase during the first week after stroke onset. However, the most interesting data arises from the comparison of the immune response between cardioembolic and atherothrombotic patients, in which the polarization towards a Th1 response is significantly greater, suggesting a pre-existing Th1-type inflammatory substrate in stroke patients affected by atherothrombotic disease. These data agree with what was observed in the atherosclerotic plaque, where CD4 + T cells express high levels of Th1-proinflammatory cytokines like IFN-γ and IL-2^19^. Furthermore, in our study we observed a parallel increase in the Th1 proinflammatory response during the first week after the ischemic event in both patients with atherothrombotic and cardioembolic strokes, probably due to the pro-inflammatory effect of ischemic tissue in the activation and in the recruitment of adaptive peripheral immunity.

The two main limitations of this study are the small sample size, at least partly due to the stringent selection of IS patients and the lack of evaluation of other lymphocyte subpopulations, in addition to CD4 + T-bet + and CD4 + GATA3 + T cells, potentially involved in the peripheral immune response in acute stroke. Another limitation of the study is the absence of the sample size analysis given the exploratory nature of the study and the absence of preliminary data.

## Conclusion

The involvement of a specific immune response in the peripheral blood of IS patients, already in the very early stages of the disease, suggests that, in addition to the pathogenetic aspect, strokes are not all equal also from an immunological point of view. The identification of the specific types of immune cells that are involved in ATHS provide a useful marker for atherothrombotic stroke, and greater insight into the biological mechanisms of recovery, possibly suggesting a future personalized immune-mediated treatment for the IS patient. Further studies, conducted on a larger cohort of IS patients and investigating the peripheral adaptive immune response in a more extensive way, are needed.

## Supplementary Information

Supplementary Figures.

## Data Availability

Access to de-identified data is available upon reasonable request through the corresponding author.
