# Supplementary material for: Th1/Th2 polarization of peripheral immune response in atherothrombotic and cardioembolic stroke: a prospective study
Source: Sci Rep. 2022 Sep 30;12:16384. doi: 10.1038/s41598-022-20515-x (PMC9525580; doi:10.1038/s41598-022-20515-x)
Supplement: Supplementary file 1 — Supplementary Figures. [file 41598_2022_20515_MOESM1_ESM.pdf]

Figure 1. CONSORT flow Diagram of the study

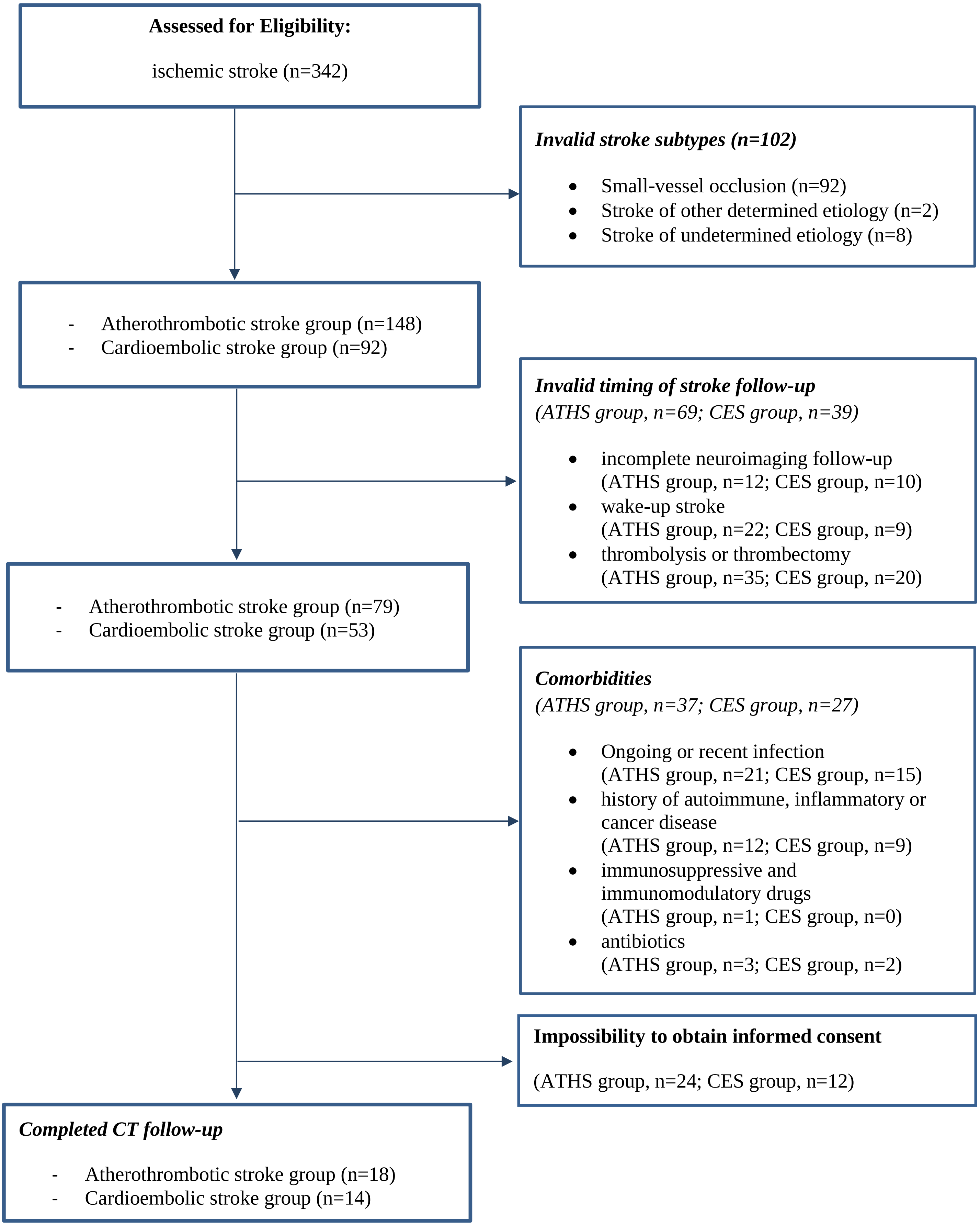

Figure legend: CONSORT flow diagram showing data on enrolment, number of patients in ATHS and CES group at different time points and causes of patient exclusion. ATHS: atherothrombotic stroke; CES: cardioembolic stroke.
